# Editorial of Special Issue “Materials for Energy Applications 2.0”

**DOI:** 10.3390/ijms24054892

**Published:** 2023-03-03

**Authors:** Sun-Jae Kim

**Affiliations:** 1 Department of Nanotechnology and Advanced Materials Engineering, Sejong University, 209, Neungdong-ro, Gwangjin-gu, Seoul 05006, Republic of Korea; sjkim1@sejong.ac.kr; 2 Metal-organic Compounds Materials Research Center, Sejong University, 209, Neung dong-ro, Gwangjin-gu, Seoul 05006, Republic of Korea

Energy is a key factor in determining the growth of human society. The production of energy and the storing of energy for a later usage is an important action in this current scenario. In recent years, many technologies involve energy storage and conversion applications. Among these, secondary batteries (Li-ion and Na-ion), supercapacitors, solar cells, electrochemical water splitting, photoelectric water splitting, and photocatalytic are most frequently concerned in these prompt technologies. In this Issue, a theoretical and experimental analysis of materials for sustainable and clean energy technologies is outlined. The construction of materials is an important factor in determining the performance of energy storage and conversion devices.

Naveenkumar et al. [1] reported on carbon-coated hetero-structured ZnS-FeS_2_ anode materials for lithium-ion batteries. The bimetal sulfide-based anode delivers a better lithium-ion battery performance than single metal sulfide. Here, heterostructure nanoarrays offer a number of advantages, such as abundant electroactive sites for redox reactions, a larger electrode–electrolyte contact surface, a shorter electrolyte diffusion path, and fascinating synergistic effects between the heterointerface. However, the achieved performance of the heterostructure is not satisfactory in Lithium-ion batteries. Moreover, the bimetal sulfide was coated with carbon using the hydrothermal method, which boosts the electrical conductivity and results in a good structural flexibility. The carbon coating and nature of carbon that exists on the surface of hetero-structured ZnS-FeS_2_ was identified from the Raman spectrum. The initial discharge capacity of the ZnS-FeS_2_@C composite delivered a capacity of 1481 mAh g^−1^ at 0.1 A g^−1^. It exhibited a capacity of 821 mAh g^−1^ at a current density of 1 A g^−1^ after the 500th cycle. This result has clearly exposed the importance of the construction of a heterostructure followed by carbon coating techniques in lithium-ion battery applications.

JH kim et al. [2] fabricated a sterilized rayon-activated carbon fiber (RACF) as an electrode material for electric double-layer capacitor applications. RACFs were prepared by the steam activation method. The effect of various time intervals of steam-activated RACFs was investigated in this study. The XRD data revealed the formation of typical isotropic carbon materials. The results showed that as the activation time increased, the structure of the pores in the RACF changed from being rich in micropores to mesopores. Even though the activation yield was high at about 28–37%, the pores in the RACF were very good, with a specific surface area of 1990–2150 m^2^/g and a mesopore fraction of 20.5–31.1% as the activation time increased. Particularly, the specific capacitance of RACF-70 was found to be higher than that of YP-50F at current densities of 2 and 50 mA/cm^2^ by about 26% and 150%, to 103.6 and 85.8 F/g, respectively. Additionally, RACFs had a low IR-drop, low charge transfer resistance, and low Warburg impedance. Finally, the RACF made by activating it with steam had better pore properties and an electrochemical performance than YP-50F. It also had the potential to function as a better electrode material for EDLC. The results of this study should make it easier to use an RACF as an effective performance-enhancing electrode material for the EDLC, which is cheap and environmentally friendly.

Dun Wu et al. [3] reported on the low-cost effective fabrication of nitrogen-doped carbon Fe_2_O_3_/carbon nanosheet nanocomposite by the laser ablation method. By including a photothermal agent of nano-magnetite into the polyimide precursor, a flat and dense Fe_3_O_4_-PI hybrid film with a good near-infrared light absorption may be created after thermal curing. It can be turned into composite electrodes of carbon nanosheets coated with nitrogen-doped Fe_3_O_4_ nanoparticles when scanned with a 1064 nm laser beam. The composite electrode provides an exceptional overpotential of 247 mV at a current density of 10 mA cm^2^ in 1M KOH due to the management of the electronic configurations of Fe-O sites in anti-spinel Fe_3_O_4_ with doped N atoms. The intriguing help of Fe_3_O_4_ octahedral Fe sites is thought to aid water dissociation via bi-molecule Volmer reaction pathways. Our established fabrication technique not only provides new insights into the simple synthesis of affordable Fe-based electrocatalysts for hydrogen production but it may also be used to develop electrode materials for energy storage and conversion applications.

KH Chung et al. [4] reported on the H_2_ production phenomenon of bismuth ferrite catalyst using liquid phase plasma. The solo-gel method was employed to prepare the bismuth ferrite catalyst. The phase and purity of the catalyst was confirmed by XRD data. The polygonal morphology was revealed by SEM and TEM images. The bandgap energy of bismuth ferrite was around 2.0 eV; it suggests that it is a good candidate for a photo-catalytic reaction in the visible region. The light absorption behavior of bismuth ferrite was well extended in the visible region. It can absorb both UV and visible light produced from the liquid phase plasma. The order of HER is bismuth ferrate > Ni/TiO_2_ > TiO_2_. The hydrogen evolution rate of bismuth ferrite in 10% methanol solution was higher than distilled water. Further, bismuth ferrate shows good catalytic behavior in the visible light region; it performs well as a feature in clean hydrogen production applications.

Metal-based electrodes have good electrochemical catalytic activity for the conversion of pollutant organic moiety to useful products. Hong Lu et al. [5] reported on the electroreduction of CO_2_ into ethanol using copper as a catalyst. In this work, the copper metal catalyst was prepared by the electrodeposition method using monoethanolamine as a regulating agent on the surface of a fluorinated tin oxide plate. The spectroscopic investigations showed that the concentration ratio of localized Cu^+^ and Cu^0^ on copper electrodes was monitored from 1.24/1 to 1.54/1 by tweaking with the help of monoethanolamine. The faradic efficiencies of ethanol and C_2_ are 48% and 77%, respectively, at a voltage of −0.6 V versus a reversible hydrogen electrode. The presence of Cu^+^ and Cu^0^ in an electrode could stabilize the formation of *CO intermediate. The intermediate could significantly involve the formation of the C-C bond towards the production of ethanol. This result exhibits the successful fabrication of electrocatalyst and their selectivity of CO_2_ reduction reactions.

We looked at how well hematite (α-Fe_2_O_3_) precipitation catalysts worked with methane (CH_4_) and found that they worked very well and stayed stable below 500 °C. [6] This study compared the structural state of hematite nanoparticles (NPs) that were formed in water from the precursor ferric chloride hexahydrate using NaOH or NH_4_OH as a precipitant at pH 11, and then heating up to 500 °C to oxidize CH_4_ at low concentrations (5% by volume in air). The decreasing conversion (%) of CH_4_ values over time was discussed in terms of how goethite and hydrohematites NP precursors change into magnetite and how the structure of calcined hydrohematites changes. After goethite loses its water, the water and vacancies that were built into it change into hydrohematite, which is different from α-Fe_2_O_3_. The surface area S_BET_ of protohematite-based Fe_2_O_3_ NH-70 precipitate was 53 m^2^/g greater than that of goethite-based precipitate. Hydrohematites with water and voids in their structure oxidized methane more efficiently.

Designing and developing catalysts with benign, low-cost, and efficient photocatalytic activity is one of the most pressing challenges in environmental remediation. Metal–organic frameworks (MOFs) are used as a template precursor to the synthesis of a variety of functional materials using various treatment approaches that result in order structures with a large surface area, regulated pore texture, and a high carbon content. Using a facile MOF-based solvothermal self-assembly approach, Maniyazagan et al. [7] successfully produced a flower-like indium oxide (In_2_O_3_-MF) catalyst. Morphology and structural research indicate that the In_2_O_3_-MF photocatalyst has a flower-like structure. In the presence of NaBH_4_ and visible light, the catalytic performance of In_2_O_3_-MF, In_2_O_3_-MR, and In_2_O_3_-MD catalysts for the reduction of 4-NP and MB degradation was analyzed. As electron-hole pairs, reactive oxygen species (ROS) such as superoxide radicals and hydroxyl ions are responsible for the photocatalytic degradation of organic pollutants. The photocatalytic reduction and degradation reactions of 4-NP and MB, accompanied by the generation of charge carriers by UV-light irradiation of a flower-like In_2_O_3_-MF photocatalyst surface, resulted in valence bond stimulation, electron transfer to the conduction band (CB), and the formation of an equal number of holes in the valence band. These photoexcited charge carriers created superoxide radicals and hydroxyl ions upon contact with dissolved oxygen (O_2_) and water molecules (H_2_O) in 4-NP and MB. Using In_2_O_3_-MF as a catalyst, they were able to reduce 4-NP by 99.32 percent in 20 minutes and MB by 99.2 percent in three minutes. After five consecutive cycles of catalytic tests, the conversion rates of catalytic 4-NP and MB remained above 95 and 96 percent, indicating that the In_2_O_3_-MF catalyst has an excellent catalytic performance and a high reutilization rate.

The most recent developments in hydrogen storage technology utilizing nanosized complex hydrides were detailed [8]. Methods for enhancing thermodynamics and accelerating sluggish kinetics have been summarized. Thermodynamic destabilization, cation and anion substitution, the eutectic formation approach, doping and additives, selecting the appropriate scaffold and electron tuning, and the now-famous nanoconfinement are all examples. The primary benefits and long-lasting drawbacks of employing such strategies were discussed in light of real-world examples found in the literature. The effective de- and re-hydrogenation behavior of the reported nanocomposites lends support to the discussed mechanistic insights. Moreover, the author says that, among the improvement pillars presented before, the electronic synergy direction is not yet fully explored and is an area where substantial advances can be foreseen based on recent developments. To achieve the long-term goal of energy storage, known as the sustainable hydrogen economy, researchers from both the theoretical and experimental spheres will collaborate.

Perovskite solar cells (PSCs) have demonstrated competitive power conversion efficiencies with the potential for a higher performance, but their stability and nonradiative recombination of carriers are still limited. To address these issues, this Special Issue introduces inverted PSCs and discusses and summarizes the latest research of inverted hybrid PSCs [9]. First, the architecture and working principle of inverted PSCs are presented. Then, the materials regarding inverted PSCs, including charge transport materials and perovskite films, are discussed. In the end, the challenges of inverted PSCs and the strategies for improving the performance are discussed.

As another way to solve the issues mentioned above, quantum dots (QDs) materials are considered promising materials for the high performance of PSCs [10]. This article showed QDs as additives in electron transport layers (ETLs), hole transport layers (HTLs), and perovskite films, and directly analyzed electron transport materials (ETMs), hole transport materials (HTMs), light-absorbing layers, and luminescent downshifting materials. Particularly, this Special Issue summarizes the role of QDs in PSCs and analyzes the perspectives and related issues.

As the global energy demands continue to rise, academia and industry are on a mission to discover renewable and innovative energy sources. Photovoltaic materials are promising candidates for clean and renewable energy technology applications. Due to the tunability of their structures and the local displacement of their B-site atoms within the original structure of these materials, pyrochlore oxides with the formula A_2_B_2_O_7_ (A_2_B_2_O_6_O′) have emerged as potential multiferroic materials. In addition to possessing the proper properties for use in TE devices, pyrochlore oxides are also effective photon absorbers. Zeesham et al. [11] studied the optical, electronic, and thermoelectric properties of the pyrochlore oxides La_2_Tm_2_O_7_ (Tm = Hf, Zr) using first-principles-based DFT calculations. Using the FP-LAPW technique, the ground-state properties of pyrochlore oxides were investigated to gain insight into their potential applications in thermoelectric devices and solar cells. Growing scientific interest in pyrochlore oxides as potential materials for renewable energy applications was the driving force behind this investigation. This family of complex materials is fascinating for applications in photovoltaic and thermoelectric devices due to their high energy conversion efficiency, which is dependent on their high absorption capacity and figure of merit. Due to the relatively wide band gaps and structural stability of La_2_Tm_2_O_7_ (Tm = Hf, Zr), these materials are promising candidates for UV photovoltaic devices. On the basis of the investigated structural properties and the ground state energy, we have determined that La_2_Hf_2_O_7_ is more stable than La_2_ZrO_7_. La_2_Hf_2_O_7_ and La_2_ZrO_7_ are direct bandgap materials with 4.45 and 4.40 eV, respectively, as shown by their energy band structures. By analyzing the TDOS spectra, it was determined that these pyrochlore oxides are nonmagnetic compounds. On the basis of ε_2_ (ω), they can conclude that these pyrochlore oxides absorb UV photons efficiently.

In this Special Issue entitled “Materials in Energy applications 2.0” under the physical chemistry of chemical physics in the *International Journal of Molecular Sciences*, our aim was successfully achieved for this topic.
